# Synthesis, Characterization and Biological Investigation of New N-Modified Spinorphin Analogs

**DOI:** 10.3390/ph15101251

**Published:** 2022-10-11

**Authors:** Petar Todorov, Stela Georgieva, Jana Tchekalarova, Subaer Subaer, Petia Peneva, Hartati Hartati

**Affiliations:** 1 Department of Organic Chemistry, University of Chemical Technology and Metallurgy, 1756 Sofia, Bulgaria; 2 Department of Analytical Chemistry, University of Chemical Technology and Metallurgy, 1756 Sofia, Bulgaria; 3 Institute of Neurobiology, Bulgarian Academy of Sciences, 1113 Sofia, Bulgaria; 4 Material Physics Laboratory, Physics Department, Universitas Negeri Makassar (UNM), Makassar 90223, Indonesia

**Keywords:** spinorphin analogs, rhodamine peptides, biological activity, anticonvulsant activity, electrochemistry, fluorimetry

## Abstract

The emergence of diverse peptide derivatives has been due to constant efforts to find a specific peptide with pronounced biological activity for effective application as a therapeutic. Spinorphin-peptide products have been reported to possess various applications and properties. In the present study, spinorphin peptides with a rhodamine residue and a modification in the amino acid backbone were synthesized by a solid-phase method using Fmoc chemistry. The results obtained from the spectral and electrochemical techniques used: Scanning electron microscopy (SEM), UV-vis, fluorescence, infrared spectroscopy (IR), and voltammetry were used to elucidate the structural characteristics and some physicochemical properties to gain insight into their behavior in the solid state and in aqueous solutions with different pHs. Both Rh-S5 and Rh-S6 had compound anticonvulsant effect comparable to Rh-S against psychomotor seizures at the highest dose of 20 μg. Furthermore, Rh-S6 showed a strong ability to inhibit seizure propagation and had a similar threshold to Rh-S against the intravenous pentylenetetrazol induced clonic seizure in mice; one of the three hybrid spinorphin analogs tested when screened for anticonvulsant activity. Biological tests against several bacterial pathogens such as *Staphylococcus aureus*, *Escherichia coli*, and *Bacillus cereus* showed similar results to negative control of the new peptide derivatives. The compounds also showed weak activity against *Candida albicans* fungus. The antioxidant testing results revealed more than 50% activity by reviewing the radical deterrence capabilities of 2,2-diphenyl-1-picrylhydrazyl (DPPH). The results are indicative of the ongoing search for universal antimicrobial agents with pronounced synergism when used simultaneously as anticonvulsant, antibacterial, and antifungal agents.

## 1. Introduction

Spinorphin or LVV-hemorphin-4 with an amino acid sequence Leu-Val-Val-Tyr-Pro-Trp-Thr is an endogenous, non-classical opioid peptide of the hemorphin family [1]. These hemoglobin-derived peptides were identified and isolated from the bovine spinal cord [2] and act as regulators of enkephalin-degrading enzymes [3]. Like other hemorphins, the heptapeptide spinorphin is an angiotensin-converting enzyme (ACE) antagonist and inhibits enkephalinases such as neutral endopeptidase (NEP), aminopeptidase N (APN), and dipeptidyl peptidase III (DPP3) [3]. Moreover, spinorphin possesses an analgesic effect for morphine-resistant pain pathways [1,4] and significantly inhibits bradykinin (BK)-induced nociceptive flexor responses [5]. Spinorphin demonstrated numerous physiological effects such as antinociceptive, antiallodynic, and anti-inflammatory properties [6].

Rhodamine B is a xanthene derivative with high lipophilicity that is often used in medicine, biology, and biotechnology as a coloring reagent in fluorescence microscopy, flow cytometry, fluorescence correlation spectroscopy, and ELISA. Its application in practice is due to its ability to bind covalently to biomolecules and thus can be used as a marker in bioassays [7,8]. Therefore, Rhodamine B derivatives are essential for studying more complex biochemical processes and activities. Such kinds of noncytotoxic fluorescence-based probes of bioactive molecules possess desirable features and excellent spectral characteristics and relatively facile syntheses [9,10,11].

Recently, Todorov et.al. synthesized and studied a series of new hemorphin peptide derivatives to find more potent anticonvulsants and analgesics with no side effects [12,13,14]. In the present study, further attempts were carried out to synthesize and fully characterize three new N-modified analogs of spinorphin with rhodamine B and their structure–property relationships in biological action examined. Given the above mentioned properties of biopeptide spinorphin and fluorescent rhodamine dye, it was expected that the newly obtained hybrid peptide compounds would possess a synergistic effect. However, no data are available in the literature or are scarce for spinorphin and its analogs. Therefore, the synthesis, characterization, neuropharmacological evaluation, acid-base properties, and electrochemical behavior of new hybrid spinorphin analogs modified at the N-terminus with rhodamine B are presented in this study with the aim to discover a new type of bioactive signaling molecule.

Spinorphin has shown a wide range of biological effects, but it is still not well developed and has few prepared synthetic analogs to date. As already mentioned, spinorphin belongs to the hemoglobin-derived peptides and, more specifically, to the non-classical opioid peptides of the hemorphin family [15,16,17]. Our group is one of the leading pioneers in designing, synthesizing, and characterizing these novel biopeptides. The synthesis and detailed survey of new analogs of spinorphin were conducted with several modifications in the peptide skeleton. The design and synthesis of different peptide analogs have led to significant changes in both peptide activity and affinity for different types of receptors or enzymes [18]. Therefore, a modification of natural spinorphin was executed via changes in its N-terminus with the fluorescent xanthene dye rhodamine B, expecting a synergistic effect in the newly synthesized hybrid peptide compounds (Rh-S, Rh-S5, Rh-S6). Since it is known that the amino acid proline is crucial for the exhibition of biological effects, a study of the structure–activity relationship was carried out by replacing natural proline with unnatural steric restricted amino acids such as 1-aminocyclopentanecarboxylic acid (Ac5c) and 1-aminocyclohexane carboxylic acid (Ac5c) (neuropeptides Rh-S5 and Rh-S6). Conformationally restricted amino acids such as Ac5c and Ac6c are increasingly used in medicinal chemistry as they stabilize the desired conformation required for biological activity [19,20]. According to Blishchenko et al., the amino acid sequence Tyr-Pro-Trp is responsible for selective binding to opioid receptors [20]. Therefore, the aim was to stabilize the desired conformation required for biological activity and evaluate the effect of proline on the biological activity of the new spinorphin analogs.

## 2. Results and Discussion

### 2.1. Chemistry

**Scheme 1:**
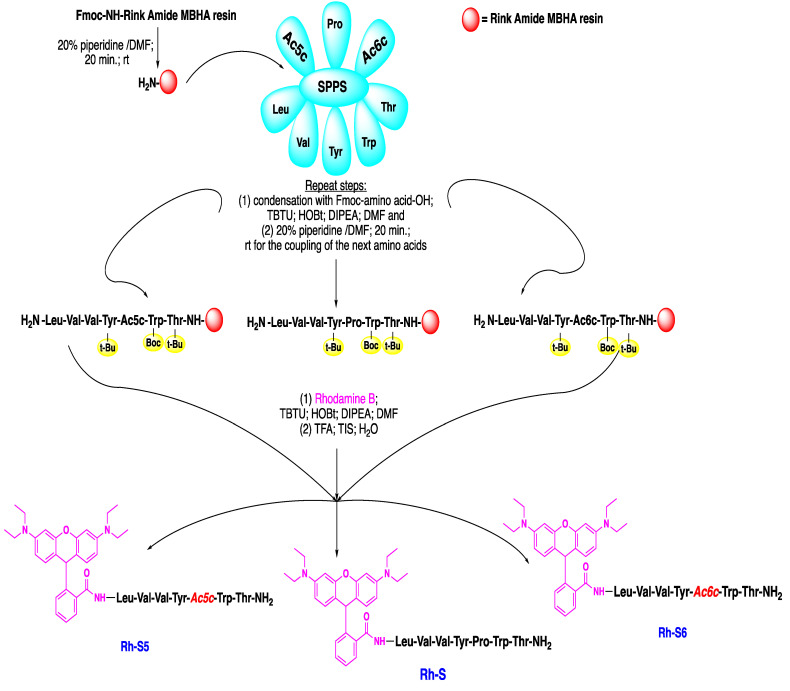
Schematic presentation of the SPPS–Fmoc chemistry of new spinorphin peptide-based analogs bearing rhodamine B side-chain (Rh-S, Rh-S5, and Rh-S6).

The synthetic route for obtaining the novel spinorphin peptides: rhodamineB-Leu-Val-Val-Tyr-Pro-Trp-Thr-NH_2_ (Rh-S), rhodamineB-Leu-Val-Val-Tyr-Ac5c-Trp-Thr-NH_2_ (Rh-S5), and rhodamineB-Leu-Val-Val-Tyr-Ac6c-Trp-Thr-NH_2_ (Rh-S6) is shown in Scheme 1. The peptides were synthesized by the most widely used and proven method in peptide chemistry, the solid-phase peptide synthesis (SPPS)-Fmoc (9-fluorenylmethoxycarbonyl) strategy. The basic concept of this procedure is the stepwise construction of the peptide chain assembled on an insoluble polymeric carrier, Rink-Amide MBHA (4-methylbenzhydrylamine) resin. The chain extension starts from the C-terminal residue. The preparation of C-terminal amides is a widely used approach in the preparation of biologically active peptide molecules, as it thus increases the resistance of peptides to enzymatic degradation [21,22,23]. The peptide formation undergoes a repetitive amidation reaction between an activated carboxyl group of one amino acid and an amino group of the other, as shown in Scheme 1. This strategy is based on the reaction between rhodamine B with the N-terminal amino group of the spinorphin analogs directly to the resin. Herein, the coupling is accomplished by activation with (2-(1H-benzotriazol-1-yl)-1,1,3,3-tetramethyluronium tetrafluoroborate) (TBTU) and hydroxybenzotriazole (HOBt) in the presence of N,N-diisopropylethylamine (DIPEA). The Fmoc group protects the amino group of all used amino acids. The Fmoc-group was easily removed with a 20% piperidine solution. The final stage of the synthesis is the cleavage of the synthesized peptides from the resin by trifluoroacetic acid (TFA), triisopropylsilane (TIS), and water.

**Figure 1:**
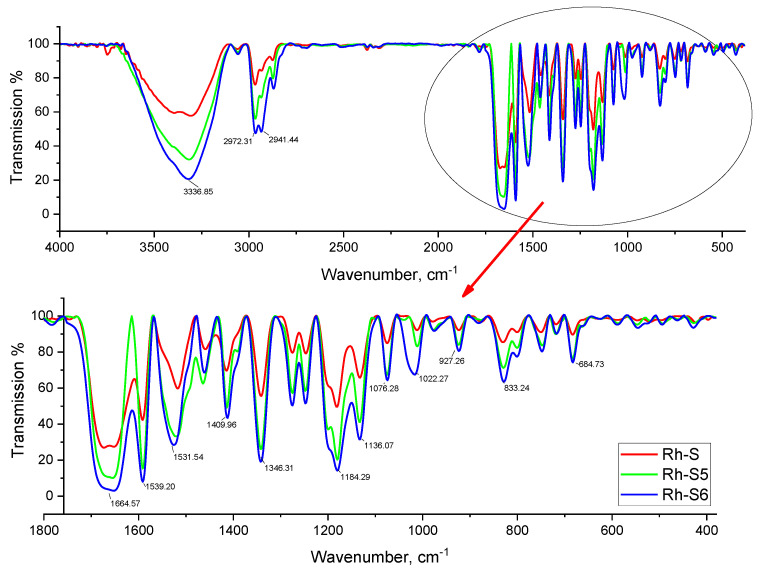
FTIR transmission spectra of Rh-S, Rh-S5, and Rh-S6.

**Figure 2:**
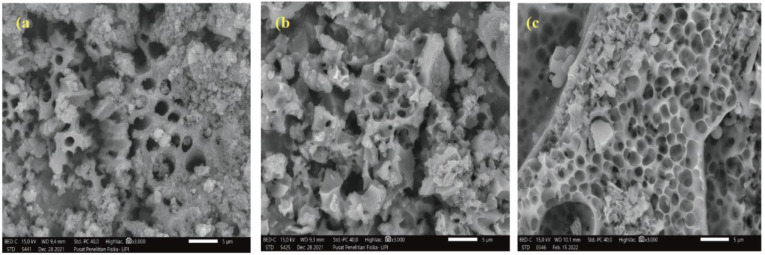
Morphological images (SEM) of the obtained rhodamine derivatives: (**a**) Rh-S, (**b**) Rh-S5, and (**c**) Rh-S6.

The three spinorphin derivatives were purified and their structures proved by appropriate modern methods and techniques described in detail in the experimental part. The IR analysis of the studied compounds showed the main vibrational characteristic lines for functional groups and bonds as follows: 3336 (N–H stretching vibration (ν_NH_)), 1664 (s) NCO (amide) stretching and 1678–1722 cm^−1^ a high-intensity peak of ν_C=O_, 1539–1531 cm^−1^ (δ_NH_) (Figure 1). The physicochemical data (*m*/*z*, t_R_, dissociation constants and isoelectric points) of the newly synthesized neuropeptide analogs are shown in Table 1. SEM analyzes of the compounds isolated in pure form were also carried out (Figure 2). The results of SEM measurement showed that the Rh peptide derivative morphology was a porous material and hence probably hydrophilic. The morphological evolution of Rh-S, Rh-S5, and Rh-S6 shows an increase in mass density and the size and distribution of pores that change drastically (Figure 2).

### 2.2. Physicochemical Characterization

#### 2.2.1. Determination of Partition Coefficient

**Figure 3:**
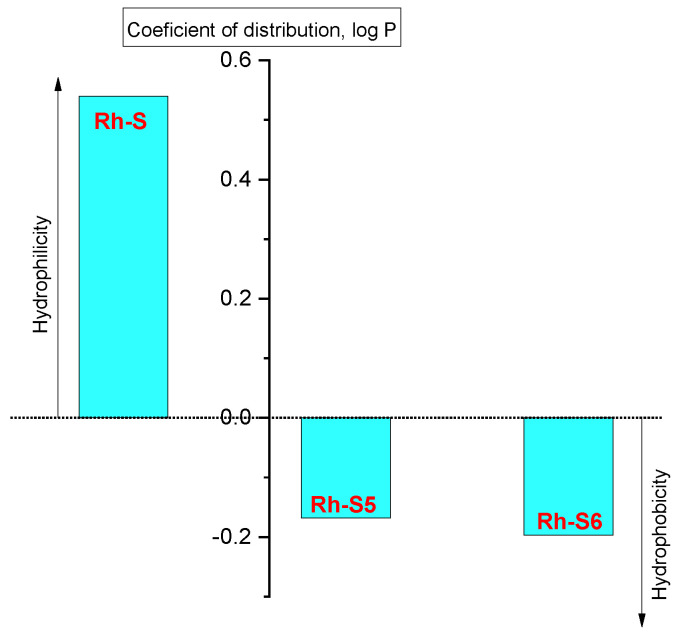
Logarithmic values of the partition coefficient (log P) of the investigated compounds: Rh- S, Rh-S5, and Rh-S6.

The effectiveness of biomolecules is mainly due to specific structural and physicochemical properties. In principle, the properties of peptides/proteins are very diverse and directly related to their bioactivity. So for example, solubility is an important factor when preparing solutions for introducing peptides into the body, as some peptides dissolve in water, others in dilute salt solutions, and there are also entirely insoluble ones, exhibiting more lipophilicity. With the manifestation of hydrophilic properties, the occurrence of a hydration process is possible; swelling, increasing their mass and volume, while it is possible to partially limit the solubility of the molecule, in which case the peptide molecule becomes more unstable. At the isoelectric point, peptides have the least ability to bind water, i.e., to hydrate; the hydration shell around the peptide molecules breaks down so that they combine to form, for example, aggregates. When the pH of the medium changes, the peptide macromolecule is charged, and its hydration capacity changes. The rhodamine peptide derivatives showed properties as neutral molecules with partition coefficient values from 0.54 to −0.2, determined by the “shake-flask method” in a 1-octanol/buffer system (pH 7.4) (Figure 3). Due to the obtained values, the studied compounds can be arranged in the following sequence from hydrophilic to hydrophobic (lipophilic): Rh-S > Rh-S5 > Rh-S6. This corresponds to the data from the morphology of the compound, where an analogous order of increasing porosity/hydrophilicity was found (Figure 3). It can be seen that the replacement of natural proline with unnatural steric restricted amino acids such as 1-aminocyclopentanecarboxylic acid (Ac5c) and 1-aminocyclohexanecarboxylic acid (Ac6c) (neuropeptides Rh-S5 and Rh-S6) leads to an increase in lipophilicity, which would assist passage through the brain barrier and increase the efficiency of the molecule. This can be seen in the study of anticonvulsant activity at the subsequent point in this manuscript.

#### 2.2.2. Determination of Acid-Base Constant (pK_a_) and Isoelectric Point (pI) by Spectral Methods

**Figure 4:**
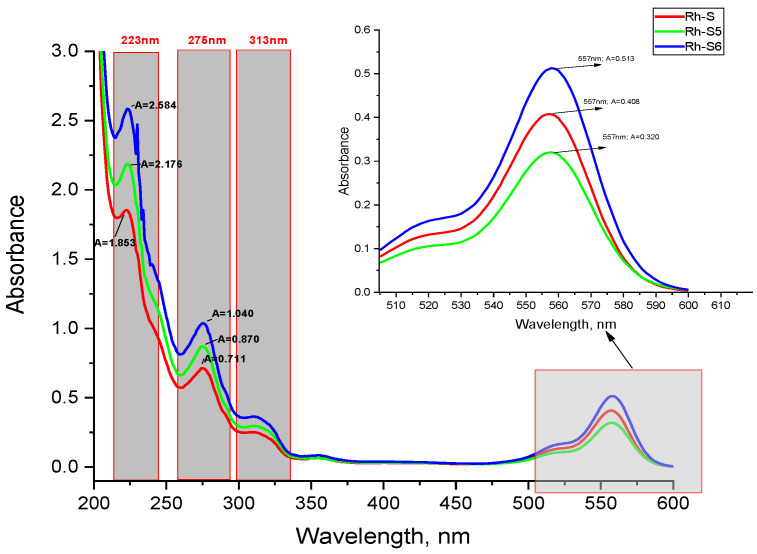
The UV–Vis absorbance spectra of equal concentration of Rh-S, Rh-S5, and Rh-S6 at pH 7.413 (phosphatic buffer solution) against water.

The amino acid sequences in the spinorphin derivatives and the rhodamine moiety show identical absorption characteristic bands in the UV–Vis region for chromophoric groups present in similar peptide compounds (Figure 4) [24]. The spectra related to the absorption of electromagnetic radiation of the spinorphin derivatives have four well-shaped absorption peaks in phosphate buffer solution, with different intensities depending on the structure of the compounds; located at λmax ≈ 233 and ≈275 nm, 315 nm and 557 nm, respectively (Figure 4). As known in the literature these are the intrinsic absorption maxima for the π→π∗ transitions of >C=O in peptide bonds (180 to 230 nm), the absorption of the π-electron systems of the aromatic groups of the indole side chains of Trp, the phenolic rings of Tyr and rhodamine (≈300 nm), and n-π∗ transitions in rhodamine (561 nm) [25,26]. The molar extinction coefficients at the long-wavelength absorption maximum are, respectively: Rh-S (ε = 2.14 × 106 cm.mol.L^−1^); Rh-S5 (ε = 2.28 × 106 cm.mol.L^−1^); Rh-S6 (ε = 3.22 × 106 cm.mol.L^−1^) (Table 2). As can be seen, the Rh-S6 exhibits the highest sensitivity.

**Figure 5:**
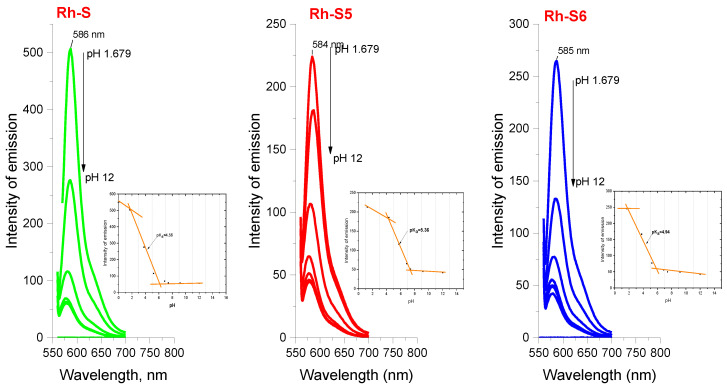
Emission spectra of spinorphin derivatives at λ^ex^ = 560 nm and different pHs. The inset plots represent I(emission)/pH obtained from the emission spectra of the respective compounds.

**Figure 6:**
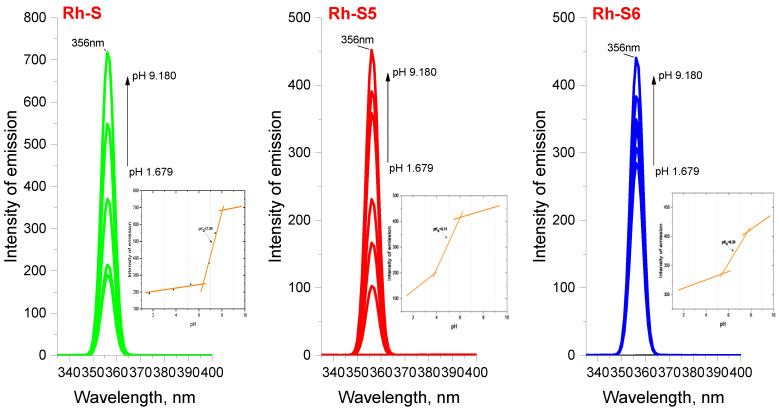
Emission spectra of spinorphin derivatives at λ^ex^ = 275 nm and different pHs. The inset plots represent I(emission)/pH obtained from the emission spectra of the respective compounds.

When studying the fluorescence properties, the emission spectra were recorded upon excitation at two wavelengths: λ^ex^ = 275 nm where tryptophan/tyrosine fluoresces, and at λ^ex^ = 560 nm where excitation of the rhodamine moiety is possible (Figure 5 and Figure 6). As can be seen, the structure of the studied compounds contains two of the known fluorescent amino acids, tryptophan and tyrosine [27,28,29]. With both amino acids present in the indicated complex mixture, the fluorescence in the 300–400 nm range is more likely to originate from the indole group of the tryptophan residue, which is known to emit at ~350 nm at an excitation wavelength of 280 nm [28]. Moreover, spectra were recorded at different pHs of the medium to determine the study compounds’ acid constants and isoelectric points. All compounds show similar behavior of fluorescence properties with minimal difference in wavelength where the compound absorbs in the visible region (1–2 nm). A strong decrease in the intensity of the emission maximum at λ^em^ = 585 nm is observed with an increase in the pH of the medium, which is due to the structural changes associated with the closure of the spirolactam ring at rhodamine [30]. pH also affects the fluorescence emission intensity of the tryptophan/tyrosine spectral line [31]. The fluorescence emission intensities of tryptophan and tyrosine were highest when the pH ranged from 6.5–7.5 and sharply decreased at pH > 7.5 [30]. In our case, we observed a sharp decrease in emission intensity occurs at pH > 9, as shown in Figure 5, which may be influenced by the presence of a rhodamine moiety. The obtained emission spectra at different pHs helped us to determine the acidity constants of the obtained compounds. pK values were obtained by selecting the inflection points of the I/pH curve at two wavelengths: 275 nm, where tryptophan emits, and 575 nm for the rhodamine part, respectively (Figure 5 and Figure 6, inserted graphs). At these points the concentrations of conjugated acid and base are equal, i.e., [HRh-S] = [Rh-S-], and according to the known relationship рН = pKa + [Rh-S-]/[HRh-S] at this point pKa = рН. Equations (1) and (2) given in the experimental part were also used to calculate the acid constants to confirm the values. Table 1 shows the average values of the constants from the repeatable results. As can be seen, the acid properties increase in the order: for pKa1: Rh-S < Rh-S6 < Rh-S5 and for pKa2: Rh-S5 < Rh-S6 < Rh-S, and in general the values for pKa2 are close to those under basic conditions and lower than those of similar rhodamine-peptide compounds [24]. The proven higher values of the constants given in [24] are due to the influence of the –OH group of tyrosine, located near the spirolactam ring of rhodamine and leading to its more effortless opening. Here, significantly, the tyrosine residue is in a more distant position, and its influence on “ring Open-ON” is attenuated. Based on the obtained values of the protolytic constants, the isoelectric points were calculated where the compounds would show zero charge and reduced solubility (Table 1). The results showed that despite the different pK values, the compounds have relatively similar isoelectric point values (~5.50), a fact that should be considered when treating them as agents with potential anticonvulsant activity when introducing them into a biological test environment. Stokes shift, showing the differences between the structure of the fluorophore in the ground S0 state and in the first excited state S1, was also determined as an essential feature for the rhodamine peptide derivatives (Figure 5, Table 2). The Stokes shift for all compounds at the rhodamine moiety is ~859 cm^−1^, which is consistent with the rhodamine derivatives known in the literature [32].

#### 2.2.3. Voltamperometric Characterization

**Figure 7:**
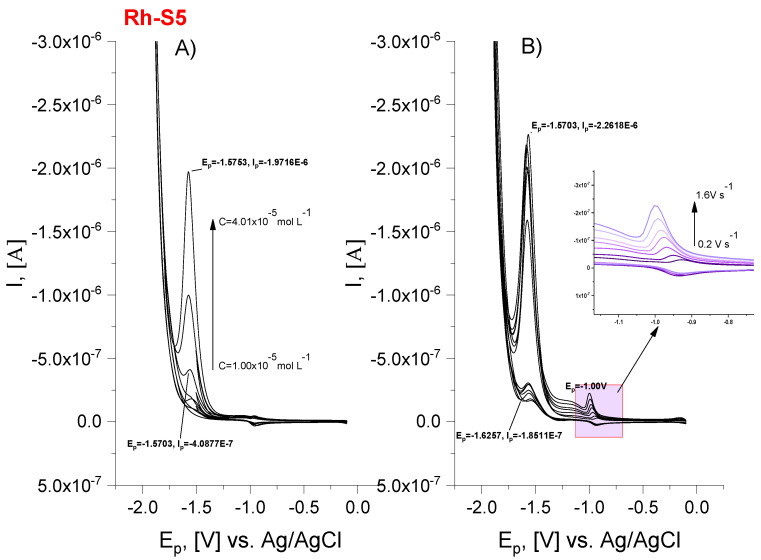
Cyclic voltammograms of spinorphin derivative (Rh-S5) in pH 6.86 phosphate buffer solution (0.1 mol L^−1^) at HMDE working electrode and Ag/AgCl, KCl as reference electrodes and (**A**) at different concentrations; (**B**) different scan rate from 0.2 to 1.60 V s^−1^.

A cyclic voltametric study to demonstrate the preservation of the integrity of the rhodamine-spinorphin molecule in contact with charged surfaces was performed in a phosphate buffer solution medium with a pH close to the physiological pH. Our study revealed that in an electrochemical environment spinorphin derivatives are reduced/oxidized leading to the production of two reduction peaks located at Ep~1.70 and −1.00 V (Table 3, Figure 7). The peak at the more negative values of the potential can be attributed to the reduction of the rhodamine part, as the difference between the potentials of the anodic and cathodic peaks is <10 mV (ΔEp = Epc − Epa) [33] as well as the ratio of peak heights: Ipc/Ipa > 1 suggesting the quasi-reversible nature of the electrode process. The reversibility of the electrode reaction is also evidenced by the fact that the current intensity increases with the increase of scan rate from 0.2 to 1.60 V.s^−1^ (Figure 7). The second peak is due to the electrochemical exchange of electrons from the amino acid sequence, most likely occurring from the tyrosine moiety. The regression equations of the function Ipc = nFACoksh (Reinmuth expression, where: Ipc is peak current, n—the number of electrons in the redox reaction of a molecule, A—the electroactive surface area, C_0_—the concentration of analyte) were used to evaluate the heterogeneous electron transfer rate constants at the rhodamine moiety. The obtained value with the order of 10^−6^ cm s^−1^ (higher than 5.0 × 10^−6^ cm s^−1^) characterizes the rhodamine reduction process as quasi-reversible (Table 4). The diffusion coefficients (D) of all compounds (Table 4) were determined using the Randles–Secik equation and the number of electrons, n = 2 [34]. The (D) values of peptide molecules varied in the sequence: Rh-S > Rh-S5 > Rh-S6 demonstrating the hypothesis that bulky molecules move at a lower speed towards the charged electrode surface.

### 2.3. Biological Analysis

#### 2.3.1. Anticonvulsant Activity

##### Six Hz Test for Psychomotor Seizures

Dose-dependent study of the activity in the 6-Hz test of new hybrid spinorphin analogs modified at the N-terminus with rhodamine B demonstrated that close to the heptapeptide Rh-S they have a protective effect against the 6-Hz-induced psychomotor seizures (Table 5). Although, Rh-S exhibited significant protection at the three doses used (5, 10, and 20 μg) (*p* ≤ 0.05 compared to control), both Rh-S5 and Rh-S6 had a significant anticonvulsant effect at the highest dose of 20 μg (*p* ≤ 0.05 compared to control).

##### Maximal Electroshock Test

Unlike the 6-Hz test, the new hybrid spinorphin analog Rh-S6 was the most potent compound against tonic seizures (Table 6). This spinorphin analog exhibited 100% protection in the MES test at the three doses studied (5, 10 and 20 μg) (*p* ≤ 0.05 compared to control) with no mortality. The other hybrid spinorphin analog RhS5 modified at the N-terminus with rhodamine also showed stronger protective potency compared to the heptapeptide Rh-S against tonic seizures at a dose of 20 µg (*p* ≤ 0.05 compared to control), suggesting that insertion of a fluorescent rhodamine dye at the N-terminus of biopeptide spinorphin increase the potency of the modified analog against generalized seizures.

##### Intravenous Pentylenetetrazole Seizure (ivPTZ) Test

**Figure 8:**
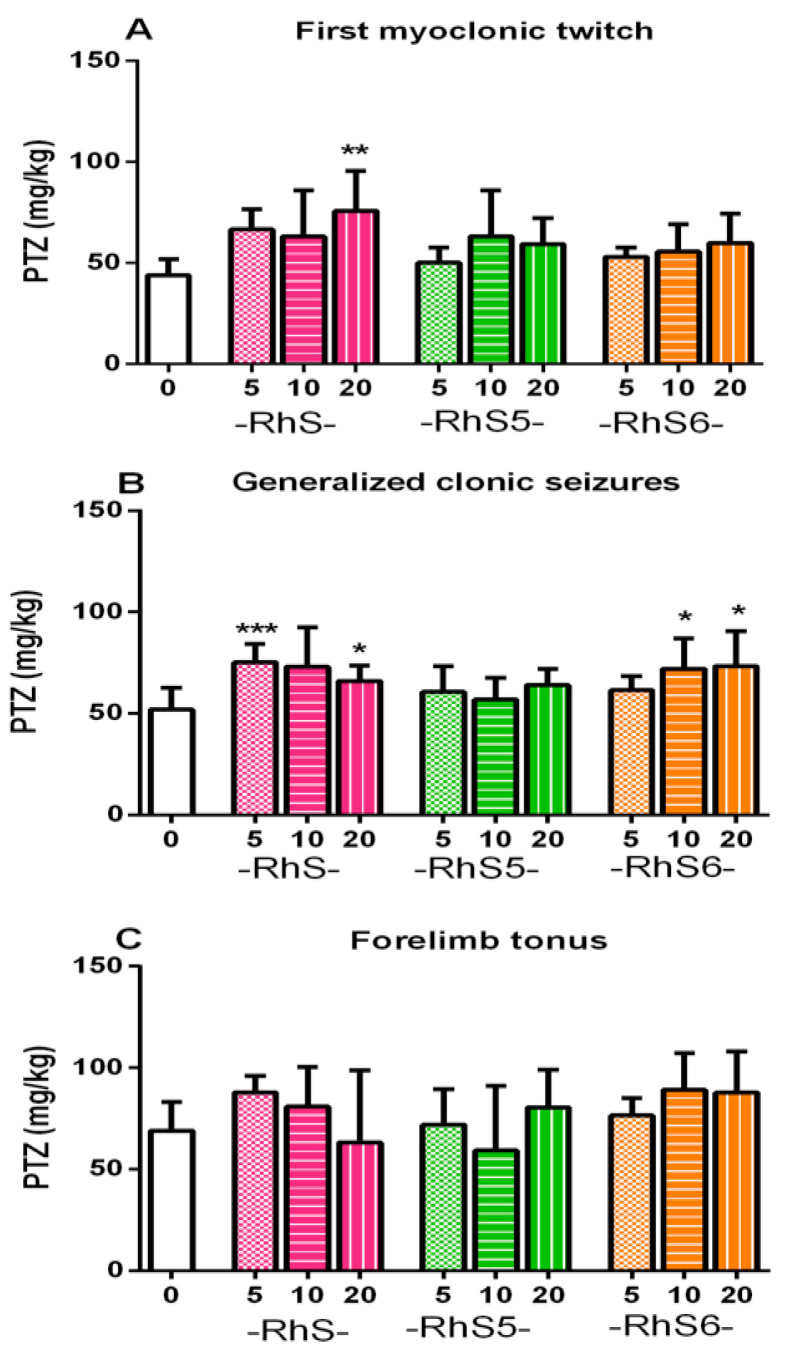
The anticonvulsant activity of Rh-S, Rh-S5, and Rh-S6 on the threshold for myoclonic (**A**), clonic (**B**), and tonic (**C**) seizures induced by the iv PTZ in mice. Statistical analyses were performed using one-way ANOVA: *p ≤ 0.05, **p < 0.01, and ***p < 0.001 compared to the control group.

The potency to elevate the seizure threshold against the three seizure phases (myoclonic, clonic, and tonic) induced by ivPTZ of the two new hybrid spinorphin analogs was explored and compared to that of the bioactive heptapeptide Rh-S. It is known that the clonic seizure is the most critical phase determining the ivPTZ-induced seizure susceptibility [35]. While insertion of rhodamine dye did not affect the seizure threshold against myoclonic and tonic seizures, it led to a similar activity to Rh-S of RhS6 against the clonic phase (*p* ≤ 0.05 compared to control) (Figure 8A–C).

#### 2.3.2. Antibacterial Activity Test of Rh-S, Rh-S5, and Rh-S6

**Figure 9:**
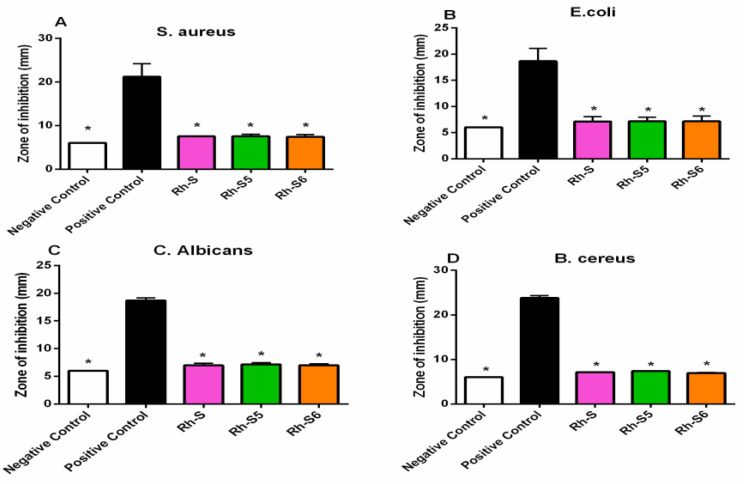
Graph of the diameter of the new peptide derivatives inhibitory zone against *S. aureus* bacteria (**A**), *E. coli* bacteria (**B**), *C. albicans* fungus (**C**), *B. cereus* bacteria (**D**), * *p* < 0.05 compared to the positive control group.

Antibacterial activity of peptide samples was carried out by using bacteria of *Staphylococcus aureus, Escherichia coli*, and *Bacillus cereus*. Based on Figure 9A,B,D the average result in the diameter of the inhibitory zone for each treatment was higher than the negative control and lower when compared to the positive control.

#### 2.3.3. Antifungal Activity Test

Antifungal activity in the new spinorphin analogs modified at the N-terminus with rhodamine B was carried out by diffusion to observe the diameter of the inhibitory zone found around the paper disc (Figure 9C). The results of antifungal activity testing with *Candida albicans* test fungi showed Rh-S, Rh-S5, and Rh-S6 peptides had almost the same antifungal activity, but slightly higher than the negative control.

#### 2.3.4. Antioxidant Activity

**Figure 10:**
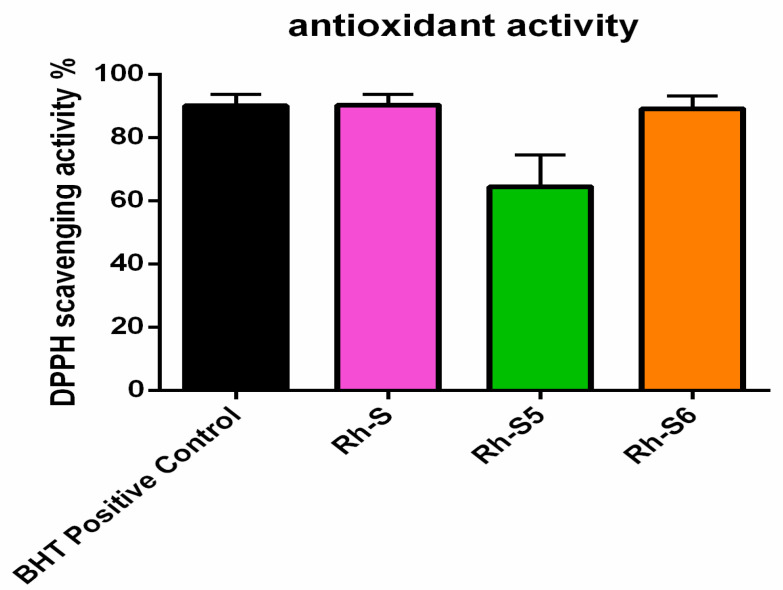
Antioxidant activity of the investigated compounds: Rh-S, Rh-S5, and Rh-S6.

The antioxidant activity of the new peptide analogs was estimated by reviewing the radical deterrence ability of DPPH (Figure 10). Compared to the positive control the investigated peptides possessed similar (Rh-S and Rh-S6) or weak (Rh-S5) activity, which was higher than 50%.

## 3. Materials and Methods

### 3.1. Synthesis

All reagents and solvents were analytical or HPLC grade and were bought from Fluka or Merck and used without further purification. The protected amino acids, 1-(9-fluorenylmethyloxycarbonylamino)-cyclopentyl-1-carboxylic acid (Fmoc-Ac5c-OH), 1-(9-fluorenylmethyloxycarbonylamino)-cyclohexyl-1-carboxylic acid (Fmoc-Ac6c-OH), and Fmoc (9-fluorenylmethoxycarbonyl)-Rink Amide MBHA (4-methylbenzhydrylamine) Resin, were purchased from Iris Biotech (Germany). Rhodamine B is from Sigma-Aldrich (Ansbach, Germany). The 3-functional amino acids were embedded as follows: Tyr as N^α^-Fmoc-Tyr(tBu)-OH, Thr as N^α^-Fmoc-Thr(t-Bu)-OH, and Trp as N^α^-Fmoc-Trp(Boc)-OH. The molecular mass and purity of the compounds were confirmed by high-resolution electrospray mass spectrometry (Thermo Fisher Scientific Inc., USA) and as well some spectral analyses were performed: IR (KBr with a Varian 660 FTIR spectrophotometer in the range 4400–600 cm^−1^), UV–Vis (Varian-Cary), SEM (SEM/FIB LYRA I XMU SEM (TESCAN), EDX detector: Quantax 200 by BRUKER), and fluorimetry (Cary Eclipse Spectrophotometry, Agilent, USA) and the detailed characteristics were previously described by us in [28].

#### General Procedure for the Peptide Synthesis of Compounds (Rh-S, Rh-S5, Rh-S6)

All N-modified peptide analogs of spinorphin were synthesized manually by the solid-phase method using Fmoc chemistry and the procedure and used reagents are given in [24]. Peptides were synthesized on Fmoc-Rink-Amide MBHA resin (loading 0.71 mmol/g resin; cross-linking 1% DVB; 100–200 mesh). Peptide chains were elongated in consecutive cycles of deprotection and coupling (see Scheme 1). The coupling reactions were performed using amino acid/TBTU/HOBt/DIPEA/resin with a molar ratio of 3/2.9/3/6/1, in a 1:1 mixture of DMF/DCM [24]. The peptides were obtained as white powders with a purity of >97% as determined by analytical HPLC. The structures were confirmed by high-resolution electrospray mass spectrometry. Peptide purity was monitored on a reversed-phase high-performance liquid chromatography (RP-HPLC), column: SymmetryShield^TM^ RP-18, 3.5 μ, (50 × 4.6 mm), flow: 1 mL/min, H_2_O (0.1% TFA)/CH_3_CN (0.1% TFA), gradient 0→100% (45 min) and 100% (5 min). The crude peptides were purified by semi-preparative HPLC on column XBridge^TM^ Prep C18 10 μm (10 × 250 mm), flow: 5 mL/min, H_2_O (0.1% TFA)/CH_3_CN (0.1% TFA), gradient 20→100% (50 min). All analytical data are summarized in Table 1.

### 3.2. Analytical Characterizations

All chemicals used were of high purity (analytical grade) and deionized water was used to prepare the solutions. Aqueous solutions of the rhodamine peptide compounds were prepared with concentrations as follows: 1.906 × 10^−3^ mol L^−1^ Rh-S, 1.405 × 10^−3^ mol L^−1^ Rh-S5, and 1.591 × 10^−3^ mol L^−1^ Rh-S6. The solutions for the physicochemical studies were prepared by appropriately diluting aliquots of the stock solutions with the indicated concentrations.

#### 3.2.1. Determination of Partition Coefficient

The partition coefficients of the spinorphin-rhodamine derivatives were determined in a 1-octanol/phosphate buffer system (NaH_2_PO_4_/Na_2_HPO_4_, pH 7.41 ± 0.01). To obtain mutual saturation in both the buffer (3 mL) and 1-octanol phases (5 mL), an amount of the test compounds (2.00 mL standard water solutions) was added to 8 mL previously vigorously stirred for 12 h at a room temperature (25 °C) distribution system. After separation, the absorbance and emission of the solutions of the two phases were measured and the amount of dissolved analyte was determined by the method of standard additions. Peptide stock solutions were used as standards to determine the analytical function coefficients. The partition coefficient was calculated by the following equation: P = C^oct/buf^/C^buf/oct^, where C^oct/buf^ and C^buf/oct^ are the molar concentrations of the solute in the mutually saturated phases of 1-octanol and buffer.

#### 3.2.2. Voltamperometric Analysis

For the voltammetric characterization of the compounds, a cyclic and differential pulse mode of a computer-controlled electrochemical system was used: a Metrohm 797 VA trace analyzer with a 797 VA stand with a three-electrode cell of a working mercury electrode, a reference electrode (Ag/AgCl, KCl (3.0 mol L^−1^) and a carbon auxiliary electrode. The measurement of signals was performed in phosphate buffer solution (0.100 mol L^−1^, pH 6.865 ± 0.1) as a supporting electrolyte and in a high-purity nitrogen atmosphere at room temperature (25 ± 1 °C). The analysis procedure was as follows: A volume of the phosphate buffer solution (7.00 mL) was added to a glass voltametric cell (50 cm^3^) and the voltametric curve was recorded to determine interference signals. Aliquots of the standard peptide solutions (50–400 µL) were sequentially added to the electrolyte solution previously degassed with high purity nitrogen for 10 min. The results presented are reported as the mean of three independent measurements.

#### 3.2.3. pK and pI Determination

Acid base constants and isoelectric points were determined fluorimetrically and confirmed by UV–Vis analyses. Absorption and emission spectra were recorded on a series of solutions with the same concentration of the peptide derivatives at different pHs (from 1 to 12). The assay data (intensity/absorbance versus pH) were processed with Origin8Pro mathematical software. The pK value were determined using the following equations:

log [(*A*_max_ − *A*)/(*A* − *A*_min_)] = *pH* − *pKa* (1)

log [(*I_F_*_max_ − *I_F_*)/(*I_F_* − *I_F_*_min_)] = *pH* – *pKa* (2)

where the minimum (*A*_min_) and the maximum (*A*_max_) absorbances are at 275 nm and 557 nm, respectively, *A* is the absorbance at the given pH value [36]; Fmax and Fmin are the maximum and minimum fluorescence emission intensity at 356 and 585 nm, respectively, *F* is the fluorescence intensity at the given pH value [37].

### 3.3. Pharmacology In Vivo Experiments

#### 3.3.1. Animals

The screeing test for anticonvulsant activity of drugs was perfomed on male ICR mice (25–30 g), purchased by the vivarium of the Institute of Neurobiology, BAS. They were kept in standard laboratory conditions and cages in groups of 5–6 per cage with ad libitum access to commercial pellets for rodents and water. After a week of adaptation, all mice were allocated in groups of 6 according to their treatment and test procedure. Each mouse was used only for one experiment. All procedures were performed in agreement with the European Communities Council Directive 2010/63/EU. The experimental design was approved by the Bulgarian Food Safety Agency (#300/№ 5888–0183).

#### 3.3.2. Drugs and Treatment

The new hybrid spinorphin analogs were dissolved in CSF (pH = 7.4) and infused intracerebroventricularly (i.c.v.) (5 μL/ventricle) at doses of 0.6; 1.2, and 2.5 μg/mouse, respectively, as described in our previous studies [38,39]. The test for anticonvulsant activity was carried out 10 min after drug administration.

#### 3.3.3. Tests for Anticonvulsant Activity

##### Six Hz Psychomotor Seizure Test

The test for assessment drug effect against psychomotor seizures was performed via a constant current device and corneal electrodes as previously [38,39]. The criterion for assessing the anticonvulsant activity was restoration of normal posture 10 s after stimulation. Controls (mice treated with vehicle) were unable to resume normal behavior and stimulation trigger different stereotypic responses and automatism such as sniffing, vibrissae, locomotion, Straub tail, or clonic seizures.

##### Maximal Electroshock Test (MES Test)

The MES test was carried out with the device used for the 6-Hz test according to the protocol reported in [38,39]. The criterion for protection was accepted when the mouse had clonic seizures. About 99% of controls injected with a vehicle exhibit tonic-clonic seizures with hind or forelimb extension.

##### Intravenous Pentylenetetrazole Seizure (ivPTZ) Test

The convulsant pentylenetetrazole (PTZ) (1%), dissolved in saline, was infused (0.005 mL/s) into a tail vein as earlier [38,39]. The activity to raise the threshold of ivPTZ-induced myoclonic, clonic, and tonic seizures and concentration, quantity of infusion (mL), and mouse body weight were recorded.

##### Test for Neurotoxicity Rotarod Test

The neurotoxicity of each compound was estimated in the rotarod test for mice according to the protocol used in previous studies [38,39].

### 3.4. Statistics

For the 6-Hz test and MES a Fisher’s exact test (two-tailed) was applied. A one-way ANOVA of variance was used for the ivPTZ test and a Dunnet post hoc test in case of significance. Statistical significance was accepted at *p* ≤ 0.05 vs. control (Fisher’s exact test). The calculations were carried out with SigmaPlot program (11.0 version) and Graph Pad Prizm (Version 7.04) (GraphPad Software, San Diego, CA, USA).

### 3.5. Test for Antibacterial Activity

To test the antibacterial activity of the peptide samples, 100 μL of bacterial spectrum (10 CFU/mL bacteria) was spread on medium nutrient agar (NA), placed onto a paper disc (6 mm in diameter), and dripped with Rh extract of 20 μL with an extract concentration of 50 mg/mL. As a positive control, we used chloramphenicol 10 μg on paper discs. DMSO 10% was used as a negative control according to peptide solvents. The treatment was repeated three times, and then the cells incubated for 24 h at a temperature of 37 °C. After that, the inhabitation of bacterial growth was carried out by measuring the diameter of the clear zone formed.

### 3.6. Antifungal Activity Test

Tests were carried out on the Candida *albicans* fungus. The concentration of the extract tested was 50 mg/mL. The culture of each test fungus was taken to be tilted using an asepticose needle and rejuvenated in a liquid medium. In each medium, there is a spore density of 10 CFU/mL. The prepared medium Sabouraud Agar (SA) in a petri dish with each culture was scratched on the top, then placed on the paper disc (paper disc), and then a peptide sample of 20 μL was placed on the paper disc. It was further incubated for 24 h and the resistance zone formed measured.

### 3.7. Antioxidant Activity

The determination of the antioxidant activity of the new peptide derivatives was carried out by inserting 77 μL of hL extract into a test tube, then adding 3 mL of DPPH solution, homogenizing, and incubating for 30 min in a dark room. The measurement of antioxidant activity was performed by a spectrophotometer with a wavelength of 517 nm. The treatment was carried out three times. The determination of activity for the standard solution was conducted by first diluting 0.025 BHT (butylated hydroxytoluene) with 1 mL of methanol. Then 3 mL of DPPH was added to 77 μL of BHT solution in a test tube, homogenized, and incubated for 30 min in a dark room. The treatment was repeated three times. For the blank, only 77 μL of methanol plus 3 mL of DPPH were used, then homogenized and incubated for 30 min in a dark room. According to Millauskas et al. (2004) [40], the free radical scavenging activity of the sample was calculated by the formula:

(3) $$\mathrm{DPPH}\;\mathrm{radical}\;\mathrm{concentration}\;(\%)=\frac{A_{\mathrm{control}}-\;A_{\mathrm{sample}}}{A_{\mathrm{control}}}\;\times \;100$$

where A_control_ is the absorbance value of the control reaction and A_sample_ is the absorbance value in the presence of the tested extracts sample.

## 4. Conclusions

Three new polar solvent-soluble spinorphin analogs modified at the N-terminus with rhodamine B were synthesized in high yields via a traditional solid-phase Fmoc strategy. All compounds are colored and soluble in physiological pH exhibiting absorptions at 557 nm. The modification in the spinorphin amino acid sequence by introducing the two unnatural steric restricted amino acids makes the excitation centers and electrochemical electron transfer more readily accessible. This was proved by an increase in the intensity of absorption and emission lines and current peaks in the spectral and voltametric characterization of the compounds. The data showed that at physiological pH the peptide derivatives were in the ionic form with increasing value of the distribution coefficient in the order: Rh-S6 < Rh-S5 < Rh-S. Based on the screening of the new hybrid spinorphin analogs modified at the N-terminus with rhodamine B in three seizure tests with a different mechanism of action in mice, we can suggest that the modification at the N-terminus with fluorescent dye rhodamine of the bioactive peptide spinorphin Rh-S6 has a strong capability to inhibit seizure spread while having comparable activity to the heptapeptide Rh-S against drug-resistant psychomotor seizures with the potency to elevate the threshold for clonic seizure. Biological tests against some bacterial pathogens such as *Staphylococcus aureus, Escherichia coli*, and *Bacillus cereus* showed that all peptide derivatives were similar to the negative control results. The compounds also showed weak activity against *Candida albicans* fungus. The results indicate the ongoing search for universal antimicrobial agents with pronounced synergism when used simultaneously as anticonvulsant, antibacterial, and antifungal agents.

## Figures and Tables

**Table 1:** Analytical data of new synthetic spinorphin peptides.

Code	Molecular Formula	^a^ [MH]^+ calculated^	^a^ [MH]^+ observed^	^b^ t_R, min_	LogP	pKa_1_	pKa_2_	pI
**Rh-S**	C_73_H_95_N_11_O_11_	1301,7213	1302,7199	41.34	0.54	7.09	4.56	5.82
**Rh-S5**	C_74_H_97_N_11_O_11_	1315,7369	1316,7357	43.21	−0.168	5.11	5.36	5.23
**Rh-S6**	C_75_H_99_N_11_O_11_	1329,7526	1330,7515	44.05	−0.197	6.35	4.94	5.65

^a^ The mass ion (MH^+^) was obtained by electrospray ionization mass spectrometry (ESI-MS); ^b^ ***t****_R_* is the retention time determined by HPLC.

**Table 2:** Photophysical characterizations of rhodamine-peptide derivatives.

Compounds	ε, [L/(mol.cm)] at 275 nm	ε, [L/(mol.cm)] at 557 nm	λ^em^ [nm] at λ^ex^ = 275 nm	λ^em^ [nm] at λ^ex^ = 560 nm	Stokes shift [cm^−1^] against λ^abs^ = 557 nm
**Rh-S**	3.73 × 10^6^	2.14 × 10^6^	356	585	859
**Rh-S5**	6.19 × 10^6^	2.28 × 10^6^	356	584	860
**Rh-S6**	6.54 × 10^6^	3.22 × 10^6^	356	585	859

**Table 3:** Voltamperometric characteristic of investigated peptides: Rh-S (1.36 × 10^−5^mol L^−1^), Rh-S5 (1.00 × 10^−5^ mol L^−1^), and Rh-S6 (1.14 × 10^−5^ mol L^−1^) at HMDE and scan rate 1.00 V s^−1^.

Compounds	E_Pc_, V	E_Pa_, V	−I_pc_, A	I_pa_, A	ΔE_pc_ = E_pc_ − E_p1/2_, mV	Nature of the Reduction Process
**Rh-S**	Ep_c1_ = −0.995 Ep_C2_ = −1.572	Ep_a2_ = −1.567	2.13 *×* 10^−7^ 1.85 *×* 10^−6^	1.85 × 10^−7^	5	QR *
**Rh-S5**	Ep_c1_ = −1.00 Ep_C2_ = −1.575	Ep_a2_ = −1.570	2.26 *×* 10^−7^ 1.97 *×* 10^−6^	1.85 × 10^−7^	5	QR
**Rh-S6**	Ep_c1_ = −1.05 Ep_C2_ = −1.595	Ep_a2_ = −1.586	2.72 *×* 10^−7^ 2.18 *×* 10^−6^	1.73 × 10^−7^	9	QR

* QR-quasi-reversible.

**Table 4:** Equation of regression of Ip_c_ vs. concentration (C, mol L^−1^); heterogen rate constant and diffusion coefficient of Rh-S, Rh-S5, and Rh-S6 peptide derivatives at HMDE electrode (with electrode area, A = 0.030 cm^2^).

Compound	Regression Equation of ip_c_, A = f(C, mol L^−1^)	k^o^_sh_ × 10^−6^ cm s^−1^	D × 10^−3^ cm^2^ s^−^^1^
**Rh-S**	Ip = 2.50 × 10^−8^+ 0.0348(±0.0023) × C, R^2^ = 0.991	6.01	3.23
**Rh-S5**	Ip = 8.03 × 10^−8^+ 0.0511(±0.0010) × C, R^2^ = 0.998	8.82	4.28
**Rh-S5**	Ip = 8.67 × 10^−8^+ 0.0533(±0.0022) × C, R^2^ = 0.995	9.21	4.34

**Table 5:** The activity of Rh-S, Rh-S5, and Rh-S6 in the 6 Hz test in mice.

Group	Dose µg/10 µL	No. of Animals Protected⁄No. of Animals Tested	% Protection
**control**	0	0/8	0
**Rh-S**	5	4/6	67 *
	10	6/6	100 *
	20	4/6	67 *
**Rh-S5**	5	1/6	17
	10	2/6	33
	20	5/6	83 *
**Rh-S6**	5	2/6	33
	10	3/6	50
	20	4/6	67 *

Statistical analysis was performed using Fisher’s exact test. * *p* ≤ 0.05 compared to control.

**Table 6:** The activity of Rh-S, Rh-S5, and Rh-S6 in MES test in mice.

Group	Dose µg/10 µL	No. of Animals Protected/No. of Animals Tested	% Protection	% Mortality
**Control**	0	0/6	0	67
**Rh-S**	5	3/6	50	50
	10	3/6	50	0
	20	4/6	67 *	33
**Rh-S5**	5	5/6	41	17
	10	5/12	42	17
	20	4/6	67 *	17
**Rh-S6**	5	6/6	100 *	0
	10	6/6	100 *	0
	20	6/6	100 *	0

Statistical analysis was performed using Fisher’s exact test. * *p* ≤ 0.05 compared to control.

## Data Availability

Data is contained within the article.
